# Medication overuse headache from a tertiary headache center in Egypt: real-world insights

**DOI:** 10.1186/s10194-025-02263-1

**Published:** 2026-01-24

**Authors:** Mona A.F. Nada, Maiar Ashraf Helmy, Marwa M. Zein, Ghada Hatem

**Affiliations:** Cairo University, Giza, Egypt

**Keywords:** Medication overuse headache, Chronic migraine, Disability, Egypt, Psychiatric comorbidity

## Abstract

**Background:**

Medication-overuse headache (MOH) is a secondary headache disorder often underrecognized in low- and middle-income countries. This study provides the first real-world data on MOH prevalence and clinical characteristics from a tertiary care center in Egypt.

**Methods:**

A cross-sectional study was conducted among 120 patients with primary headaches recruited from the Headache Unit and general outpatient clinics. Patients were classified according to ICHD-3 criteria as MOH or non-MOH. Clinical characteristics, risk factors, medication use, disability using the validated Arabic version of Migraine Disability Assessment questionnaire (MIDAS) and Headache Impact Test (HIT-6), quality of life using Arabic version of Short-Form 12 Health Survey (SF-12), and psychiatric comorbidity were assessed.

**Results:**

The prevalence of MOH was 65.8%. Chronic migraine was the most common underlying primary headache (80.5%). MOH patients were predominantly female (86.1%) and older, and were less educated than non-MOH patients. Risk factors significantly associated with MOH included insomnia (38% vs. 17.1%), musculoskeletal pain/fibromyalgia (43% vs. 19.5%), and physical inactivity (89.9% vs. 75.6%). The most overused medications were ergot derivatives (88.5%) and triptans (59.3%). MOH patients reported significantly higher headache frequency (mean 24.4 ± 7.1 vs. 11.2 ± 7.6 days/month, *p* < 0.001), greater disability (MIDAS mean 46.5 ± 28.9 vs. 19.3 ± 22.9, *p* < 0.001), and poorer quality of life (PCS 43 vs. 49.4; MCS 35 vs. 44, *p* < 0.01). Depression, anxiety, and stress scores were also significantly higher among MOH patients.

**Conclusions:**

MOH is alarmingly prevalent among Egyptian tertiary care patients, especially women with chronic migraine, emphasizing the need for national strategies on over-the-counter analgesic control and structured withdrawal programs.

## Introduction

Medication-overuse headache (MOH) is a disabling secondary headache disorder characterized by frequent use of acute headache medications, which paradoxically increases headache frequency. It represents a major factor in the transformation of episodic migraine or tension-type headache into their chronic forms and is associated with considerable socioeconomic burden [1]. Globally, the prevalence of MOH is estimated at 0.5–7.2% in the general population but may reach 30–70% among patients treated at tertiary headache centers [2]. According to the Global Burden of Disease, it has been ranked 20th among all medical conditions in terms of years lived with disability (YLDs) [3]. The burden is particularly pronounced in regions where over the counter (OTC) analgesics are easily accessible, public awareness is low, and drug regulation is limited [4]. These conditions are characteristic of Egypt, where OTC ergots and NSAIDs are inexpensive and frequently self-administered. Despite this, epidemiological data and research on MOH in Egypt remain scarce.

The present study aimed to determine the prevalence of MOH among patients with primary headache disorders in a major Egyptian tertiary center and to describe their demographic and clinical features, risk factors, medication overuse patterns, and the impact of MOH on disability, quality of life, and psychiatric comorbidity.

## Methodology

A cross-sectional observational study was conducted at the Kasr Al-Ainy Headache and Related Disorders Unit (KAHDU) and the General Neurology Outpatient Clinics at Cairo University between June 2023 and June 2024. Kasr Al-Ainy Hospitals, Cairo University, function as a tertiary referral center for patients from Cairo and neighboring governorates, providing specialized neurological and headache services to a broad and diverse population (Urban and rural).

**Fig. 1:**
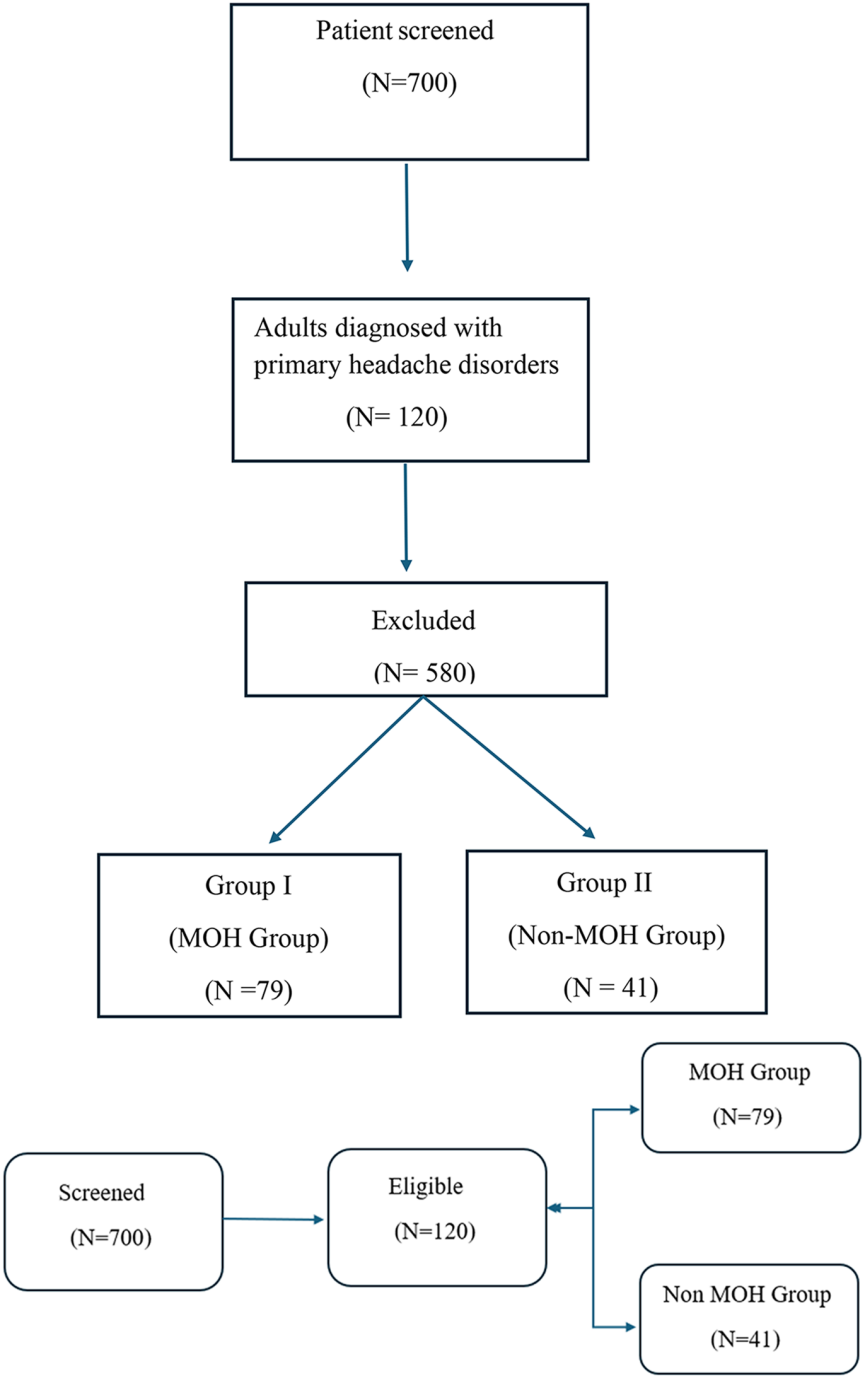
Strobe flow chart for patients included in the present study

A total of 700 patients were screened, where 120 adults (aged > 18 years) were diagnosed with primary headache disorders (17.1%) (migraine or tension-type headache) according to the International Classification of Headache Disorders, 3rd edition (ICHD-3) were enrolled, the rest of patients (580 patients) were excluded according to exclusion criteria. Patients were categorized into two groups (Fig. 1):

- Group I (MOH Group): Included 79 patients diagnosed with medication-overuse headache (MOH).
- Group II (Non-MOH Group): Included 41 patients diagnosed with primary headache disorders (migraine or tension-type headache) without MOH.

### Inclusion criteria

- Patients fulfilling diagnostic criteria for MOH or primary headache (migraine or tension-type headache) according to ICHD-3.
- Age > 18 years.
- Both sexes.

### Exclusion criteria

- Other Secondary headache disorders.
- Patients previously diagnosed with psychiatric illnesses. This was intended to allow unbiased symptom- level screening
- Alcohol or other drug addiction.
- History of detoxification treatment of MOH.

### Sample size

Sample size was calculated based on the primary objective to detect the prevalence of medication overuse headache (MOH) among patients with primary headache disorders in a tertiary center in Egypt, and as per reviewing the published articles of the prevalence of MOH. It was found that MOH among all types of headache was ranged; from 1% to 7% [5–9]. So, we calculated the sample size based on the expected 30% prevalence of MOH among primary headache patients, this prevalence was called by an expert in headache management in Egypt. The total number of patients with diagnosed primary headache at Kasralainy headache and general neurology clinics is around 160 in 4 months (expected duration for data collection). Based on the previous inputs the minimum sample size was calculated to be 120 patients to achieve 80% power, 5% significant with 10% non-response rate using Open Epi online sample size calculator.

All participants underwent a comprehensive clinical evaluation. Detailed history taking included demographic and clinical data such as age, sex, education, occupation, marital status, comorbidities, family history, and lifestyle habits. Headache characteristics were recorded, including age at onset, frequency, duration, presence of aura, and headache location. Medication use patterns were documented, including the type, dose, number of acute medications, and number of medication-use days per month. Each participant received a full general and neurological examination. The impact of medication-overuse headache on disability, quality of life, and psychiatric comorbidity was evaluated using the validated Arabic versions of standardized instruments. Headache-related disability was assessed with the Arabic validated version of Migraine Disability Assessment (MIDAS) [10] and Headache Impact Test-6 (HIT-6) [11], while quality of life was measured using Arabic validated version of the Short Form-12 Health Survey (SF-12) [12]. Headache severity was determined using the Visual Analogue Scale (VAS) [13]. Psychiatric comorbidity was assessed with the Arabic version of the Beck Depression Inventory (BDI), the Depression Anxiety Stress Scales (DASS, and the Hamilton Anxiety Rating Scale (HARS). Neuroimaging was performed when clinically indicated. The study was approved by the Cairo University Ethics Committee (MS-202-2023), and written informed consents were obtained from all participants.

### Statistical analysis

All the data collected were revised for completeness and logical consistency. The data was extracted from the google form to Microsoft Office Excel Software Program, 2019, then was transferred and analyzed into the Statistical Package of Social Science Software program, version 23 (SPSS) for statistical analysis. Continuous variables were tested for normality using the Kolmogorov Smirnov test, which revealed that the data wasn’t normally distributed, so it was summarized as median (IQR). Categorical variables were summarized as frequency and percentage (%). Group comparisons used the Chi-square for categorical variables and the Mann–Whitney U test for continuous variables. P of ≤ 0.05 was considered significant.

Binary logistic regression was performed to identify independent predictors of the MOH. Odds ratios (ORs) with 95% confidence intervals (CIs) were reported, and statistical significance was set at *p* ≤ 0.05.

## Results

### Prevalence and distribution of primary headache disorders

**Fig. 2:**
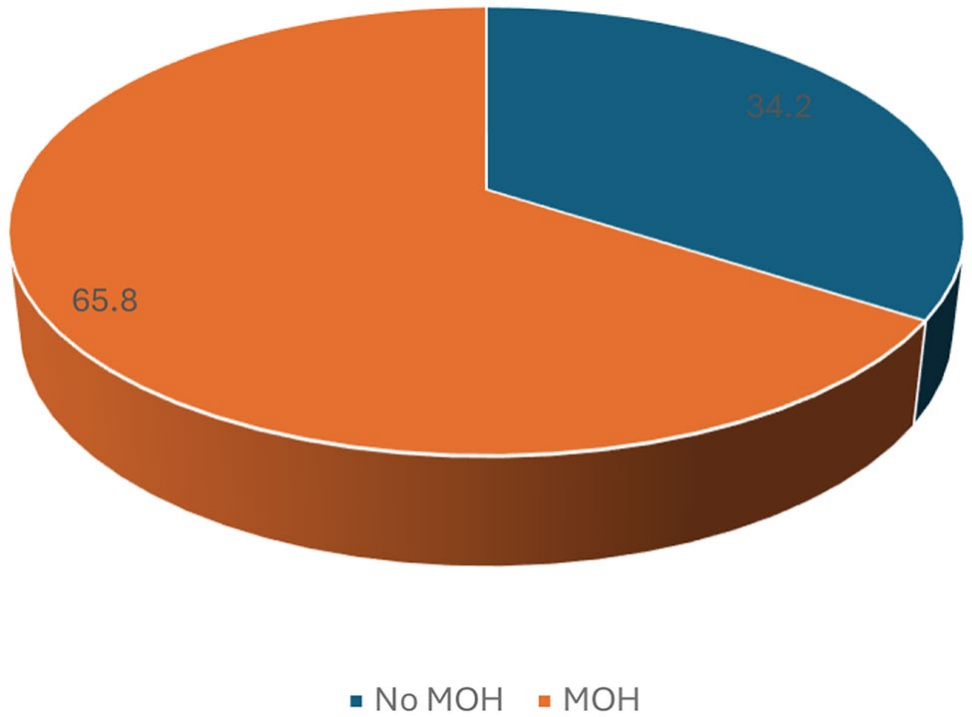
Prevalence of MOH

Of the 120 patients with primary headache disorders included in the study, the one-year prevalence of MOH was 65.8% as shown in Fig. 2. Among MOH patients, chronic migraine was the most frequent underlying primary headache type, affecting 64 patients (81.0%), followed by chronic tension-type headache in 8 patients (10.1%) and a combination of chronic tension and chronic migraine in 7 patients (8.9%).

### Clinical characteristics

The median age of the MOH group was 34, with IQR: 26–41, compared to median age of 28, with IQR: 22–39. Females predominated in both groups, representing 86.1% of the MOH group and 68.3% of the non-MOH group.

Sociodemographic characteristics (education, occupation, marital status) are summarized in Table 1, and potential risk factors in Table 2. In brief, patients with MOH were less educated and more often unemployed compared with non-MOH patients.

### Risk factors

A positive family history of headache was more common among MOH patients (78.5% vs. 51.2%). Physical inactivity was highly prevalent in both groups, but significantly higher among MOH patients (89.9% vs. 75.6%). Comorbid conditions such as insomnia (38% vs. 17.1%) and musculoskeletal pain or fibromyalgia (43% vs. 19.5%) were also significantly more frequent in the MOH group. Hypertension and diabetes were relatively uncommon in both groups (Table 2).

### Headache characteristics

In the MOH group, the median age at onset of headache was 25, with IQR 17, 32, and the median duration of headache was 5 years, with IQR 2–10 years. The frequency of headache attacks was markedly higher among MOH patients (median 30, IQR 20–30 days/month) compared with the non-MOH group (median 10, IQR 6, 15 days/month, p value < 0.001 0). The median duration of individual headache episodes in MOH patients was 12 h with IQR of 7, and 24 h ± 8.2 h.

On the visual analogue scale (VAS), 12.7% of MOH patients reported the pain as the worst possible, 67.1% as very severe, and 20.3% as moderate to severe. Headache characteristics, including type, location, and presence of aura, are detailed in Table 3. Chronic migraine was by far the most common headache subtype among MOH patients (80.5%), whereas episodic migraine predominated among non-MOH participants (61%).

### Pattern of medication use

**Fig. 3:**
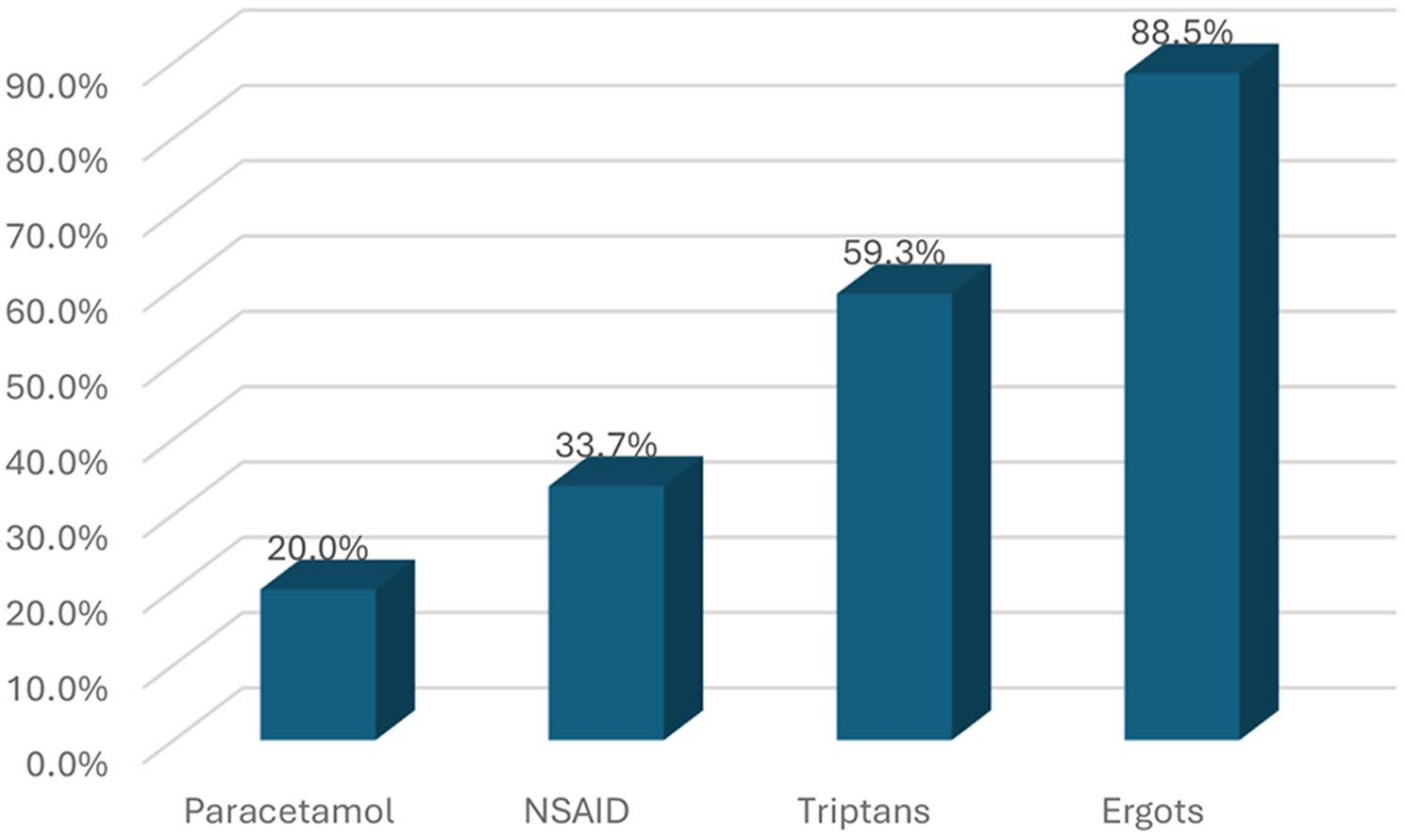
Pattern of overused abortive medications

Among the MOH group, ergot derivative (Ergotamine tartrate (1 mg)) was the most frequently overused abortive medication (88.5%), followed by triptans (59.3%), NSAIDs (33.7%), and paracetamol (20%) as shown in Fig. 3. Nearly half of the patients (49.4%) reported overuse of two abortive medications, while 36.7% used three. Regarding preventive therapy, 41 patients (51.9%) in the MOH group were on prophylactic treatment: 19 (24.1%) used a single agent, 15 (19%) used two agents, and 7 (8.9%) used three. The most frequently prescribed prophylactics were amitriptyline (35.4%), propranolol (26.6%), cinnarizine (24.1%), and topiramate (22.8%), while valproate (2.5%) was the least used.

### Comparative results between the MOH and the non-MOH groups

MOH patients reported significantly more frequent headache days per (median of 30 with IQR of 20 to 30 vs. median of 10 and IQR o 6 to 15, *p* < 0.001), though the pain severity did not differ significantly between the two groups.

Risk factors that were significantly associated with MOH included insomnia, musculoskeletal pain/fibromyalgia, and physical inactivity (*p* = 0.019, 0.010, and 0.038, respectively). No significant differences were found for other factors such as smoking, hypertension, or diabetes.

### Disability, quality of life, and psychiatric comorbidity

**Fig. 4:**
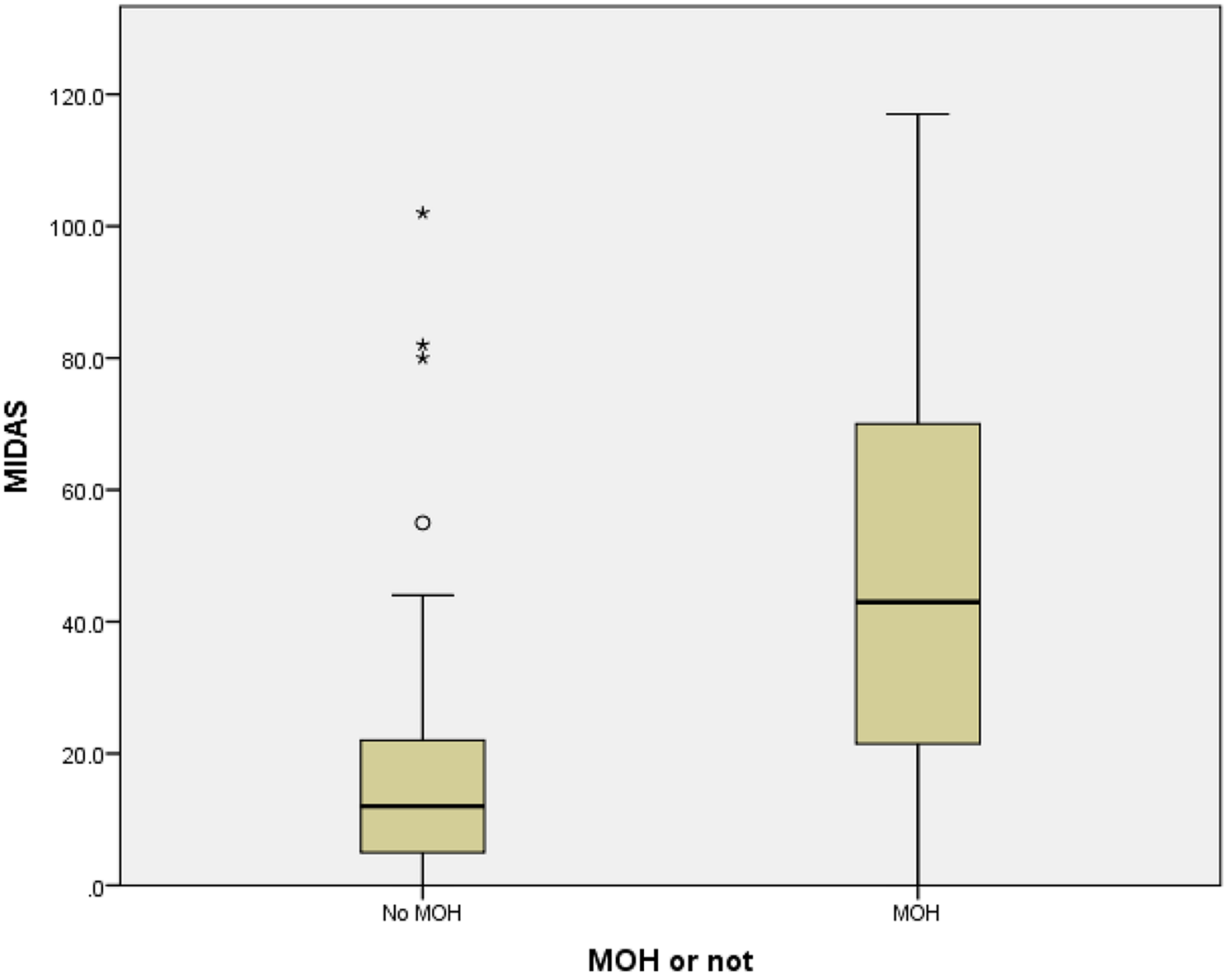
Box plot of MIDAS score between the MOH group and the non MOH group

MOH patients demonstrated significantly greater disability compared with non-MOH participants. The median MIDAS score was 43 with IQR of 21 and 70 versus 12 with IQR of 5 and 22 (*p* < 0.001), with 78.5% of MOH patients classified as severely disabled compared to 31.7% in the non-MOH group, Figure 4. Although median HIT-6 score was slightly higher among MOH patients (65 (IQR 60 and 63) vs. 61 (IQR 57 and 66), this difference did not reach statistical significance (*p* = 0.063).

Regarding quality of life, the MOH group had significantly lower mean SF-12 Physical Component Score (PCS: 43 (IQR 36,51) vs.50 (IQR 47,56), *p* = 0.001) and Mental Component Score (MCS: 34 (IQR 28,43) vs. 47 (IQR 38, 52), *p* < 0.001), indicating poorer physical and mental well-being (Table 4).

### Psychiatric assessment

Patients with MOH showed markedly higher scores for depression, anxiety, and stress across all assessment tools (Table 5). The median BDI score was 17 (IQR 9,26) in the MOH group compared to 9 (IQR 4,17) in the non-MOH group (*p* < 0.001). Similarly, DASS subscales revealed significantly higher median depression (15 (IQR 6, 25) vs. 7 (IQR 3,15), *p* = 0.003), anxiety (12 (IQR 8,22) vs. 7 (IQR 3,10), *p* < 0.001), and stress (17 (IQR 11,26 vs. 9 (IQR 3,7), *p* < 0.001) among MOH patients. The Hamilton Anxiety Rating Scale (HARS) also showed significantly greater median anxiety severity in the MOH group (18 (IQR 9,27) vs. 11 (IQR 3,19), *p* = 0.001). Across all instruments, the MOH group had a higher proportion of participants in the moderate-to-severe and extremely severe categories compared with non-MOH patients.

### Generalisability of findings

The title and abstract havebeen appropriately revised to reflect data from a tertiary care center. A final check of theQ1Q2Discussion is recommended to ensure that prevalence statements consistently refer to “tertiarycare headache patients in Egypt,” rather than implying nationwide estimates.

#### Logistic regression analysis of factors associated with MOH

Logistic regression analysis revealed that participants with a positive family history had significantly higher odds of MOH (OR = 7.54, 95% CI: 2.01–28.27, *p* = 0.003). Triggering symptoms were also a strong predictor (OR = 29.42, 95% CI: 2.05–421.37, *p* = 0.013). Moreover, headache frequency was independently associated with increased odds of the MOH, with each additional headache per month increasing the odds of MOH by 26.5% (OR = 1.27, 95% CI: 1.16–1.38, *p* < 0.001) illustrated in Table 6.

**Table 1:** Education, occupation and marital status of both groups

Characteristics	MOH patients (*N* = 79)	Non-MOH patients (*N* = 41)
**Education**		
Uneducated	19 (24.1%)	2 (4.9%)
Primary	13 (16.5%)	3 (7.3%)
Preparatory	3 (3.8%)	4 (9.8%)
Secondary	7 (8.9%)	5 (12.2%)
Diploma	16 (20.3%)	8 (19.5%)
University	21 (26.6%)	19 (46.3%)
**Occupation**		
Not Working	55 (69.6%)	15 (36.6%)
Working	24 (30.4%)	26 (63.4%)
**Marital status**		
Single	19 (24.1%)	16 (39%)
Married	57 (72.2%)	24 (58.5%)
Divorced	2 (2.5%)	1 (2.4%)
Widowed	1 (1.3%)	0 (0%)

Abbreviation: MOH: Medication Overuse Headache

**Table 2:** Risk factors among both groups

Risk Factors	MOH group *N* = 79	Non-MOH group *N* = 41
**A. Family History**		
Positive	62 (78.5%)	21 (51.2%)
Negative	17 (21.5%)	20 (48.8%)
**B. OCP/Injectable Contraception**		
No Family Planning	31 (45.6%)	16 (59.3%)
OCP	12 (17.6%)	3 (11.1%)
Injectables	7 (10.3%)	2 (7.4%)
IUD	18 (26.5%)	6 (22.2%)
**C. Special Habits of Medical Importance**		
**Physical Activity**		
Physically inactive	71 (89.9%)	31 (75.6%)
Physically Active	8 (10.1%)	10 (24.4%)
**Smoking**		
Non-Smokers	71 (89.9%)	36 (87.8%)
Smokers	8 (10.1%)	5 (12.2%)
**D. Co-morbidities**		
**Sleep Problems**		
No	49 (62.0%)	34 (82.9%)
Yes	30 (38%)	7 (17.1%)
**Muscular Pain/Fibromyalgia**		
No	45 (57%)	33 (80.5%)
Yes	34 (43.0%)	8 (19.5%)
**Diabetes Mellitus (DM)**		
No	77 (97.5%)	40 (97.6%)
Yes	2 (2.5%)	1 (2.4%)
**Hypertension (HTN)**		
No	71 (89.9%)	40 (97.6%)
Yes	8 (10.1%)	1 (2.4%)

Abbreviations: DM: Diabetes Mellitus, HTN: Hypertension, OCP: Oral Contraceptive Pills, IUD: Intrauterine Device

**Table 3:** Headache characteristics among both groups

Characteristic	MOH group (*N* = 79, %)	Non-MOH group (*N* = 41, %)
**Type of Headache**		
Episodic Tension Headache	0 (0%)	3 (7.3%)
Chronic Tension Headache	8 (10.1%)	3 (7.3%)
Episodic Migraine	0 (0%)	25 (61%)
Chronic Migraine	64 (80.5%)	10 (24.4%)
Chronic Tension and Migraine	7 (8.9%)	0 (0%)
**Site of Headache**		
Alternating	13(16.5%)	6 (14.7%)
Bitemporal	35 (44.3%)	17 (41.5%)
Frontal	1 (1.3%)	1 (2.4%)
Frontotemporal	13 (16.5%)	8 (19.5%)
Holo-cephalic	12 (15.2%)	8 (19.5%)
Occipital	4 (5.1%)	1 (2.4%)
Periorbital	1 (1.3%)	0 (0%)
**Aura**		
No Aura	32 (40.5%)	23 (56.1%)
Basilar	4 (5.1%)	0 (0%)
Sensory	9 (11.4%)	3 (7.3%)
Visual	29 (36.7%)	12 (29.3%)
Visual/Sensory	5 (6.3%)	3 (7.3%)
**Aggravating Factors**		
Absent	12 (15.2%)	5 (12.2%)
Present	67 (84.8%)	36 (87.8%)
**Relieving Factors**		
Absent	7 (8.9%)	2 (4.9%)
Present	72 (91.1%)	39 (95.1%)
**Severity using Visual Analogue Scale**		
Moderate to Severe Pain	16 (20.3%)	14 (34.1%)
Very Severe Pain	53 (67.1%)	23 (56.1%)
Worst Pain Possible	10 (12.7%)	4 (9.8%)

**Table 4:** Comparison between both groups as regards MIDAS, HIT6 and SF12 scales

Characteristic	MOH group	Non MOH group	*p* Value
**MIDAS**			< 0.001*
Mean ± SD	46.5 ± 28.9	19.3 ± 22.9	
Median (Q1, Q3)	43 (21, 70)	12 (5,20)	
**HIT-6**			0.063
Mean ± SD	63.5 ± 7.7	60.9 ± 8.2	
Median (Q1, Q3)	65 (60, 68)	62 (57, 66)	
**HIT6 Categories**	**(N = 79**,** %)**	**(N = 41**,** %)**	0.331
Little or No Impact	2 (4.9%)	1 (1.3%)	
Some Impact	5 (12.2%)	8 (10.1%)	
Substantial Impact	6 (14.6%)	6 (7.6%)	
Severe Impact	28 (68.3%)	64 (81.0%)	
**SF-12 (PCS-12) Physical Score**			0.001*
Mean ± SD	43 ± 10	49.4 ± 8	
Median (Q1, Q3)	43 (36, 51)	50 (47, 56)	
Above average physical health	25 (31.6%)	21(51.2%)	0.036*
Below average physical health	54 (68.4%)	20 (48.8%)	
**SF-12 (MCS-12) Mental Score**			< 0.001*
Mean ± SD	35 ± 11	44 ± 9	
Median (Q1, Q3)	34 (28, 43)	47 (38, 52)	
Above average mental health	11 (13.9%)	14 (34.1%)	0.01*
Below average mental health	68 (86.1%)	27 (65.9%)	

Abbreviations:** SD**: Standard Deviation, **IQ**: interquartile median, * statistically significant p value < 0.05., **MIDAS**: Migraine Disability Assessment Scale, **HIT6**: Headache Impact Test-6, **SF12**: Short Form 12, **MCS**: Mental Component Score, **PCS**: physical component score

**Table 5:** Comparison between both groups regarding depression, anxiety and stress

Characteristic	MOH Group	Non-MOH Group	*p* Value
**Beck Depression Inventory**			
Mean ± SD Median (IQR)	18.6 ± 11.2 17 (9, 26)	11.1 ± 8.7 9 (4, 17)	< 0.001*
**Beck Depression Inventory subscales**	**Number (%)**	**Number (%)**	
Normal	22 (27.8%)	22 (53.7%)	0.033*
Mild mood disturbance	14 (17.7%)	8 (19.5%)	
Borderline clinical depression	9 (11.4%)	5 (12.2%)	
Moderate depression	16 (20.3%)	4 (9.8%)	
Severe depression	15(19.0%)	2 (4.9%)	
Extreme depression	3 (3.8%)	0 (0%)	
**DASS Depression**			
Mean ± SD Median (IQR)	16.1 ± 10.7 15 (6, 25)	10.5 ± 9.7 7 (3, 15)	0.003*
**DASS Depression subscales**	**Number (%)**	**Number (%)**	
Normal	27 (34.2%)	21 (51.2%)	0.018*
Mild	8 (10.1%)	9 (22.0%)	
Moderate	18 (22.8%)	4 (9.8%)	
Severe	9 (11.4%)	5 (12.2%)	
Extremely severe	17 (21.5%)	2 (4.9%)	
**DASS Anxiety**			
Mean ± SD Median (IQR)	13.9 ± 7.7 12 (8, 22)	8.1 ± 6.8 7 (3, 10)	< 0.001*
**DASS Anxiety subscales**	**Number (%)**	**Number (%)**	
Normal	14 (17.7%)	21 (51.2%)	< 0.001*
Mild	13 (16.5%)	9 (22.0%)	
Moderate	22 (27.8%)	3 (7.3%)	
Severe	8 (10.1%)	6 (14.6%)	
Extremely severe	22 (27.8%)	2 (4.9%)	
**DASS Stress**			
Mean ± SD Median (IQR)	18.6 ± 9.9 17 (11, 26)	11.6 ± 9.6 9 (3, 7)	< 0.001*
**DASS stress subscales**	**Number (%)**	**Number (%)**	
Normal	26 (32.9%)	24 (58.5%)	0.021*
Mild	15 (19%)	9 (22%)	
Moderate	15 (19%)	2 (4.9%)	
Severe	13 (16.5%)	5 (12.2%)	
Extremely severe	10 (12.7%)	1 (2.4%)	
**HARS**			
Mean ± SD Median (IQR)	18.3 ± 11.1 18 (9, 27)	11.3 ± 9.7 11 (3, 19)	0.001*
**HARS subscales**	**Number (%)**	**Number (%)**	
No anxiety	3 (3.8%)	6 (14.6%)	0.009*
Mild anxiety	32 (40.5%)	23(56.1%)	
Mild to moderate anxiety	18(22.8%)	8(19.5%)	
Moderate to severe anxiety	26(32.9%)	4(9.8%)	

Abbreviations:** DASS Depression**: Depression Anxiety Stress Scales - Depression Subscale, **DASS Anxiety**: Depression Anxiety Stress Scales - Anxiety Subscale, **DASS Stress**: Depression Anxiety Stress Scales - Stress Subscale, **HARS**: Hamilton Anxiety Rating Scale, **IQR**: interquartile range, * statistically significant p value < 0.05

**Table 6:** Logistic regression analysis of factors associated with the MOH

Variable	B	SE	Wald	*p*-value	OR (Exp(B))	95% CI for OR
Family history (yes vs. no)	2.020	0.675	8.965	0.003	7.537	2.009–28.274
Triggering symptoms (yes vs. no)	3.382	1.358	6.199	0.013	29.416	2.054–421.368
Frequency of headache per month	0.235	0.044	29.130	< 0.001	1.265	1.162–1.378
Constant	–8.020	2.009	15.940	< 0.001	—	—

## Discussion

Medication-overuse headache (MOH) is a disabling and often underrecognized secondary headache disorder that contributes significantly to the chronification of primary headaches and imposes substantial social and economic burdens, particularly in low-resource regions such as Africa [14]. A self-perpetuating cycle of escalating medication use and increasing headache frequency underlies its pathophysiology [15].

In this study, the prevalence of MOH among tertiary care headache patients in Egypt instead of the sentence between brackets (Egyptian tertiary center patients) reached 65.8%, markedly exceeding global estimates of 0.5–7.2% in the general population [1, 16] and aligning with rates reported in specialized centers, where Evers and Marzinia reported also that in a tertiary care and headache referral centers, the prevalence of MOH accounted for 30% of headache patients in Europe and 50% of headache patients in the United States [17]. The widespread availability of cheap OTC ergots and NSAIDs, along with pharmacist-advised self-medication, likely explains this difference. Thus, reflecting the need for awareness of the patients about the need for medical consultation, avoiding self-medications and excess use of OTC analgesics and ergots.

In the present study, chronic migraine was the predominant primary headache associated with MOH, a finding consistent with international observations. Irimia et al. 2011 [18] reported that migraine was the most frequent primary headache linked to MOH, affecting 67.5% of patients. Similarly, Shand et al. 2015 [19] found that more than 80% of individuals with MOH had migraine as the underlying headache disorder, while tension-type and post-traumatic headaches accounted for the remaining cases.

The female predominance observed among MOH patients in the current study is consistent with global trends and align with Bigal et al. 2004 [20] similarly reported that 76% of MOH patients were women. Most studies have shown that MOH is significantly more common in females, with a male-to-female ratio of approximately 1:3–4, particularly among middle-aged adults (30–50 years) [19, 21–24]. This sex disparity may be partly explained by hormonal influences [25] and psychosocial factors, as women often report higher levels of stress and anxiety, which can aggravate migraine, increase reliance on acute medications, and ultimately predispose to MOH [26].

In the present study, MOH was significantly more prevalent among less educated and unemployed patients, consistent with prior evidence linking lower socioeconomic status to chronic headache disorders. Limited education and unemployment may contribute to reduced healthcare access, underdiagnosis, and greater reliance on self-medication. Moreover, unemployment can disrupt daily routines, including sleep and dietary habits, and increase psychological distress such as anxiety and depression—all factors that may exacerbate headache frequency and medication overuse [27]. Low educational attainment has been identified as an independent risk factor for MOH [23], and several studies have demonstrated a consistent association between MOH and lower education levels, although it remains unclear whether this reflects a causal relationship or results from the disabling impact of chronic headaches [22, 24, 28]. These findings reflect the negative association of unemployment and lower educational levels on MOH, lower educational levels are associated with lack of awareness for the need of medical consultation for headaches and consequently excessive use of medications, that lead to MOH for headache relief, leading to financial and medical burden.

Regarding risk factors for MOH, this study found that MOH patients had statistically significantly higher rates of insomnia, muscular pain or fibromyalgia, and physical inactivity compared to non-MOH patients. These identified risk factors align with population-based studies, reinforcing the biopsychosocial model of MOH. Those results agree with a longitudinal population-based cohort study that used data from the Nord-Trøndelag Health Surveys, which found that there is a risk for developing MOH among individuals who had chronic musculoskeletal complaints, and that physical inactivity doubled the risk of MOH. There is evidence supporting the presence of central sensitization amongst subjects with chronic complaints, psychological predisposition (lack of pain-coping ability), and possibly shared susceptibility genes [29]. This could point to the need for awareness of the importance of physical activity, proper sleep hygiene, proper dietary habits, management of muscular pains and managing all risk factors for MOH. Among Egyptian population physical inactivity and improper sleep hygiene are common due to lack of time due to major commitments and due to cultural backgrounds.

Logistic regression analysis revealed that a positive family history of headaches, headache frequency and the presence of headache triggering factors are strong predictors for developing MOH, this came in line with Cevoli who reported that 28.7% of MOH patients had family history for chronic headaches [30], this points to the need for screening family members of patients with chronic headache and teaching them when to seek medical advice and when and how to use abortive medications and when they will need prophylactic medications to avoid the development of and the negative impact of chronic headache and MOH and to detect the triggering factors and to try to avoid the as much as the patients can to decrease the frequency of headaches, risk of developing MOH and improving the quality of life.

Compared with the MOH group, the MOH group experienced a statistically significant increase in headache attacks per month, with no difference in headache severity. This partially agrees with previous studies, which reported that MOH patients had significantly more monthly headache days (MHDs), higher severity, and higher pain intensity scores [31, 32]. The high frequency of headache attacks per month could be related to stressful life events either related to work, relationships or financial issues, also due to environmental and poor dietary habits. Because of the frequent headache attacks patients tend to take NSAIDs or ergot derivatives by their own, without medical prescription, to be able to perform their duties, also due to cultural background that headache is not a serious issue requiring medical consultation.

Regarding the pattern of medication overuse, ergot derivatives (Ergotamine tartrate (1 mg)) was the leading cause of MOH in our cohort, contrasting with data from Europe and the United States, where triptans and NSAIDs are most implicated. This discrepancy likely reflects Egypt’s unique medication environment, characterized by the widespread availability, low cost, and over-the-counter accessibility of ergot preparations. Our findings are consistent with Sarchielli et al. 2012 [33], who reported that ergot-containing and caffeine-based compounds increase the risk of headache chronification, particularly in patients with high baseline headache frequency. In contrast, Schwedt et al. 2018 [34] found that MOH patients were more likely to overuse triptans, while ergot use was rare. Similarly, other studies have shown a higher prevalence of MOH related to analgesic overuse compared with triptans or ergots [29, 35]. These differences likely stem from regional variations in drug accessibility and prescribing practices. In Egypt, ergot derivatives remain inexpensive, readily available without prescription, and widely perceived as highly effective for migraine relief, encouraging frequent self-medication and contributing to overuse.

In the current study, most MOH patients used more than one abortive medication simultaneously. This aligns with findings by Zidverc-Trajkovic et al. 2007 [36], who reported that 45% of MOH patients overused two or more abortive medications, while 55% overused a single drug class. In their study, NSAIDs were the most overused agents, followed by combination analgesics, whereas ergotamine compounds were overused alone in 6.7% of patients and in combination with other medications in 36.1%. The widespread practice of providing pharmaceuticals without a physician prescription, despite the considerable health and legal concerns involved, presents significant issues for pharmacy control in Egypt. People frequently go to a pharmacy to get antibiotics, sedatives, or even hormonal drugs, and they get them right away without a prescription or any kind of medical supervision. Despite several regulations that forbid the dispensing of medications over-the-counter have been enforced by the Ministry of Health and Population. The usefulness of those rules on improper dispensing, however, is not well supported.

Self-medication is a global health issue that is fluctuating and expanding among various populations worldwide. As expected, it is more prevalent in developing countrie. It ranges from 11.20% to 93.70% depending on the population and country. Adherence to the proper practical guidelines and restrictions for the use of SM saves time and money and lessens the load on medical services. However, improper use in the form of unnecessary conditions, improper doses, and/or duration of intake is more common. Such practices may lead to irrational drug use, delayed seeking of medical advice, drug interactions, and increased risk of adverse drug events, or serious events such as antimicrobial drug resistance [37].

Several studies have highlighted the strong association between MOH and affective disorders. Sarchielli et al. 2016 [38] reported that 28.4% of MOH patients had depression and 22.7% had moderate-to-severe anxiety, while the MOTS trial found that 37% experienced significant anxiety and 74% had depressive symptoms [39]. Similarly, the COMOESTAS study reported depression in 39.2% and anxiety in 49.5% of MOH patients [40]. Our results are consistent with these findings, showing significantly higher depression, anxiety, and stress scores in MOH compared with non-MOH patients. This speculates the association of depression and anxiety with MOH, despite few studies studying this association in MOH but there are many studies reporting this association in chronic migraine and chronic daily headaches. Therefore, it is important to assess depression, anxiety and stress in MOH patients for the proper management of both conditions thus stopping this vicious circle, reducing the suffering of the patients and improving their quality of life.

The Eurolight study [41] and a large U.S. survey [34] also confirmed this link, emphasizing the bidirectional relationship between chronic headache and psychiatric distress. Proposed mechanisms include cephalophobia leading to medication overuse [40], amygdala hyperexcitability from chronic analgesic exposure [29], and stress-related activation of the hypothalamic pituitary adrenal axis [42]. Notably, mood and anxiety symptoms often improve following successful medication withdrawal [40].

According to a study conducted in many centers in Italy, 28.4% of patients with medication overuse headache were found to have a mood disorder or depression, and that 22.7% had moderate to severe anxiety based on their scores on the Beck Depression and Anxiety Inventory, respectively [38]. Furthermore, about 37% of individuals participating in the MOTS trial exhibited symptoms of moderate to severe anxiety and higher proportion of individuals with depression, with 74% having a patient health questionnaire (PHQ-9) score of at least 5 [39]. In the COMOESTAS study, 39.2% of patients were found to have depression, and 49.5% had anxiety, determined by a hospital anxiety depression score (HADS) of at least 8 [40].

As chronic migraine was the most prevalent primary headache in our MOH patient group, they showed significantly more severe depression than those with chronic tension MOH patients. Our findings were consistent with the Eurolight trial, a cross-sectional survey that recruited adult populations aged 18–65 years from 10 European Union countries and discovered a high association between MOH and psychiatric problems, and that migraine patients with MOH were found to have a significant association with depression and anxiety [41].

In a nationwide survey of adults with migraine in the United States, 39.5% (832 out of 2107) of patients who excessively used medications for headache showed symptoms that indicated the presence of anxiety and/or depression. These results illustrate the connections between anxiety, depression, and MOH [34].

Although there is a high probability that individuals with chronic migraines and medication overuse headaches may have symptoms of anxiety and sadness, the underlying link between chronic migraines and MOH, anxiety, and depression remains ambiguous. Possible psychological symptoms, such as fear from the occurrence of a severe migraine attack known as cephalophobia, may lead to an increased frequency of taking acute migraine medication [40]. Also, chronic use of analgesics leads to increased neuronal excitability in the amygdala complex, which may explain the occurrence of anxiety and depression in MOH [29]. The symptoms of depression and anxiety can be diminished after effectively treating MOH [40]. The activation of the sympathetic nervous system and hypothalamic-pituitary axis, as well as the disruption of activity in various brain regions involved in the pain matrix and cognitively affective phenomena, are the putative mechanisms by which stress causes headaches, chronicity, and transformation to MOH [42].

In order to improve and guarantee the health and well-being of the Egyptian people, the Egyptian health care system must overcome several obstacles. In addition to fighting diseases linked to poverty and illiteracy, the system also must deal with new diseases and illnesses brought on by the contemporary urban lifestyle. The population’s demands for more and better care as well as cutting-edge medical technology are rising because of increased access to international communications and commerce. Egypt’s healthcare system is quite diverse, with a wide range of governmental and private providers and agents for financing. Currently, organizations in Egypt oversee, fund, and deliver health care in all three economic sectors: private, parastatal, and governmental.

## Conclusion

Medication overuse headache is highly prevalent among Egyptian headache patients, particularly women with chronic migraine. Overuse of ergots and triptans, combined with OTC availability, contributes substantially. MOH is associated with marked disability, poor quality of life, and psychiatric comorbidities. Urgent interventions in Egypt should include public education, regulation of OTC sales of ergots and NSAIDs, and early preventive therapy.

### Limitation

1. The main limitation of the present study is that it was conducted at a tertiary referral center (Kasr Al-Ainy Hospitals, Cairo University), which may have introduced selection bias, as patients with more severe or chronic headache disorders are likely to be overrepresented. However, this limitation is partly mitigated by the fact that Kasr Al-Ainy is the largest tertiary center in Egypt, serving patients from Cairo and multiple surrounding governorates, thus providing a relatively broad representation.
2. The sample size, although adequate for group comparisons, may not capture the full regional variability in medication use patterns across Egypt, particularly given the lack of prior national data on MOH prevalence and characteristics.
3. Another limitation is the lack of follow-up data to assess the impact of MOH detoxification on headache frequency, chronicity, disability, and associated psychiatric symptoms such as depression and anxiety. This was out of the scope of this study.

### Strengths

- This study represents the first comprehensive assessment of MOH among Egyptian headache patients, addressing a major gap in regional data.
- It was conducted at Kasr Al-Ainy Hospitals, Cairo University, the largest tertiary referral center in Egypt, serving patients from Cairo and multiple surrounding governorates, which enhances the representativeness of the sample.
- The study used validated Arabic versions of key assessment tools (MIDAS, HIT-6, SF-12, BDI, DASS, and HAM-A), ensuring cultural and linguistic reliability.
- A multidimensional evaluation was performed, encompassing demographic, clinical, pharmacological, disability, quality-of-life, and psychiatric parameters, providing a comprehensive understanding of MOH in this population.

## Data Availability

The datasets used and/or analyzed during the current study are available from the corresponding author on reasonable request.

## Abbreviations

BDI: Beck Depression Inventory
DASS: Depression Anxiety Stress Scales
HADS: Hospital anxiety depression score (HADS)
HARS: Hamilton Anxiety Rating Scale
HIT-6: Headache Impact Test-6
ICHD-3: International Classification of Headache Disorders, 3rd edition
KAHDU: Kasr Al-Ainy Headache and Related Disorders Unit
MCS: Mental Component Score
MHDs: Monthly headache days
MIDAS: Migraine Disability Assessment
MOH: Medication-overuse headache
OTC: Over the counter
PCS: Physical Component Score
PHQ-9: Patient health questionnaire (PHQ-9)
SF-12: Short Form-12 Health Survey
VAS: Visual Analogue Scale
YLDs: Years lived with disability
